# Identifying pathogenic variants in the Follistatin‐like 1 gene (*FSTL1*) in patients with skeletal and atrioventricular valve disorders

**DOI:** 10.1002/mgg3.567

**Published:** 2019-02-05

**Authors:** Stuti Prakash, Andrea Mattiotti, Marc Sylva, Barbara J. M. Mulder, Alex V. Postma, Maurice J. B. van den Hoff

**Affiliations:** ^1^ Department of Medical Biology Amsterdam UMC, location AMC Amsterdam The Netherlands; ^2^ Department of Cardiology Amsterdam UMC, location AMC Amsterdam The Netherlands; ^3^ Department of Clinical Genetics Amsterdam UMC, location AMC Amsterdam The Netherlands

**Keywords:** Fstl1, gene expression, heart development, skeletal abnormalities

## Abstract

**Background:**

Follistatin‐like 1 (Fstl1) is a glycoprotein expressed throughout embryonic development. Homozygous loss of Fstl1 in mice results in skeletal and respiratory defects, leading to neonatal death due to a collapse of the trachea. Furthermore, Fstl1 conditional deletion from the endocardial/endothelial lineage results in postnatal death due to heart failure and profound atrioventricular valve defects. Here, we investigated patients with phenotypes similar to the phenotypes observed in the transgenic mice, for variants in *FSTL1*.

**Methods:**

In total, 69 genetically unresolved patients were selected with the following phenotypes: campomelic dysplasia (12), small patella syndrome (2), BILU (1), and congenital heart disease patients (54), of which 16 also had kyphoscoliosis, and 38 had valve abnormalities as their main diagnosis. Using qPCR, none of 69 patients showed copy number variations in *FSTL1*. The entire gene body, including microRNA‐198 and three validated microRNA‐binding sites, were analyzed using Sanger sequencing.

**Results:**

No variants were found in the coding region. However, 8 intronic variants were identified that differed significantly in their minor allele frequency compared to controls. Variant rs2272515 was found to significantly correlate (*p* < 0.05) with kyphoscoliosis.

**Conclusion:**

We conclude that pathogenic variants in *FSTL1* are unlikely to be responsible for skeletal or atrioventricular valve anomalies in humans.

## 1 INTRODUCTION

Approximately 3%–4% of all life‐born babies have a congenital malformation. In a minority of these cases, the underlying cause is identified as a chromosomal abnormality, a familial or de novo pathogenic variations, or as an environmental cause (e.g., pollution, alcohol abuse), or a combination of these conditions (Kuciene & Dulskiene, 2008; Liu et al., 2009). However, in the vast majority of congenital abnormalities, the cause is unknown.

We have recently prepared a global and conditional knockout of Follistatin‐like 1 (*Fstl1*) (Prakash et al., 2017; Sylva et al., 2011) which both show congenital abnormalities (see below). FSTL1 is a secreted glycoprotein (OMIM 605547), belonging to SPARC family of proteins (Sylva, Moorman, & Hoff, 2013), and is a potential prognostic marker for cardiovascular disease (Widera et al., 2012, 2009). *FSTL1* expression is induced by TGFβ and thought to be an extracellular regulator of BMP signaling (for review, see (Mattiotti, Prakash, Barnett, & Hoff, 2018; Sylva et al., 2013)). The human *FSTL1* gene is located on chromosome 3q13.33 and comprises 11 exons. Exons 2 to 11 encode the 308 amino acid long protein. The last exon also contains the coding sequence for microRNA‐198 (*MIR198*). As a consequence, the *FSTL1* primary transcript serves as mRNA or pre‐miRNA in a mutually exclusive way (Hinske, Galante, Kuo, & Ohno‐Machado, 2010; Sundaram et al., 2013, 2017). Moreover, multiple microRNA‐binding sites are present in the 3’UTR of *FSTL1,* of which three (miR‐206, miR‐32–5p, and miR‐27a) have been shown to be functional and suppress *FSTL1* expression (for review, see: (Mattiotti et al., 2018)). Thus far, no human congenital disease has been associated with alterations in *FSTL1*. In the analysis of the exomes of over 120,000 individuals, no homozygous loss‐of‐function variations were observed, and heterozygous ones have only been described in twenty‐five individuals (http://gnomad.broadinstitute.org/gene/ENSG00000163430).

Homozygous knockout (*Fstl1* KO) mice are born in normal Mendelian ratios but suffocate within hours due to the inability to breathe as a consequence of the absence and/or hypoplasia of tracheal cartilage rings. These KO mice show extensive skeletal defects, including abnormal spine curvature, bending of long bones, displacement of the atlas, the absence of the patella, dislocation of the hip and digit defects as well as cardiac, lung and ureter abnormalities (Geng et al., 2011; Sylva et al., 2011; Xu et al., 2012). The phenotype of *Fstl1* KO mice is comparable to a rare genetic disorder known as Campomelic Dysplasia (CD; OMIM 114290) (Mansour, Hall, Pembrey, & Young, 1995). In the majority of CD cases, the underlying cause is a loss of function of one *SOX9* allele (OMIM 608160) (Corbani et al., 2011). However, in approximately 5% of CD cases, no underlying genetic cause can be identified (Unger, Scherer, & Superti‐Furga, 1993). Other rare genetic syndromes that show (partial) overlap with the phenotype of *Fstl1* KO mice are small patella syndrome (SPS; OMIM 147891) (see Table 3) (Offiah et al., 2002), caused by heterozygous loss‐of‐function pathogenic variants in *TBX4* (OMIM 601719) (Bongers et al., 2004), and BILU (OMIM 609296) (B‐cell Immunodeficiency, Limb anomalies, and Urogenital malformations) (see Table 3) (Edery et al., 2001; Hugle et al., 2011). The finding that *Fstl1* KO mice display both abnormal spine curvature and respiratory distress at birth can also point toward kyphoscoliosis, a musculoskeletal disorder characterized by an abnormal curve of the spine that causes extrapulmonary restriction of the lungs that further results in impairment of pulmonary functions (Pajdzinski et al., 2017). In addition, these patients often have psychological problems (for example, anxiety, difficulty in performing daily activities) which are also observed in mice, in which *Fstl1* is genetic deleted from the small dorsal root ganglion neurons (Li et al., 2011).

Besides the full *Fstl1* knockout, a set of conditional knockouts has also been described. Cardiomyocyte‐specific deletion of *Fstl1* using αMHC‐Cre (Shimano et al., 2011) or fibroblasts specific deletion using S100A4‐Cre (Tanaka et al., 2016) resulted in viable offspring with no reported developmental defects. However, conditional ablation of *Fstl1* from the endocardial/endothelial lineage using Tie2‐Cre (*Fstl1* endoKO) results in neonatal lethality between 2 and 4 weeks after birth (Prakash et al., 2017). These *Fstl1* endoKO mice showed profound cardiac and valve defects, including an enlarged stressed heart and long and thick atrioventricular valves, displaying a myxomatous phenotype and being dysfunctional (Prakash et al., 2017). The *Fstl1* endoKO mice also have cardiac conduction abnormalities including fragmented QRS and increased QT intervals. Besides the cardiac phenotype, the lungs are also affected showing impaired remodeling of the small pulmonary vasculature (Tania et al., 2017).

This study aimed at identifying whether variations in *FSTL1* underlie human congenital abnormalities that show phenotypic similarity with those that are observed in the *Fstl1* KO and/or *Fstl1* endoKO mice. To this end, we selected 69 patients with genetically unresolved campomelic dysplasia, small patella syndrome, BILU syndrome, kyphoscoliosis and/or defects of the mitral or tricuspid valves, and Sanger sequenced the *FSTL1* gene body and determined the copy number.

## 2 MATERIAL AND METHODS

### 2.1 Editorial policies and ethical considerations

This study was approved by the Medical Ethical Committee at the Academic Medical Center in Amsterdam. Written informed consent was obtained from all participants.

### 2.2 Patient recruitment

Genomic DNA was obtained of 12 patients suffering from unexplained campomelic dysplasia, that is, no pathogenic variants for *SOX9*, two patients suffering from unexplained small patella syndrome, that is, no pathogenic variants for *TBX4*, and one patient suffering from BILU syndrome. Furthermore, from the CONCOR repository (van der Velde et al., 2005), which comprises DNA of more than 16,000 adult patients with a congenital abnormality, 54 patients were selected with an unexplained congenital abnormality, comprising kyphoscoliosis and/or cardiac valve defects. Anonymized samples were analyzed for variations in the coding sequence of *FSTL1* (NM_007085.5) by using Sanger sequencing and for copy number variation using genomic qPCR.

### 2.3 Sequence analysis of *FSTL1*

Using the primers listed in Table 1, we PCR amplified the *FSTL1* coding region of the exons and surrounding regions which are considered the splice donor and acceptor sites. We included, furthermore, the parts of the 3’UTR that code the validated miRNA‐binding sites (*MIR206/32–5p/MIR‐27a*) and miRNA (*MIR198*). After amplifications, the PCR product was diluted and sequenced using Big Dye Terminator (BDT). The analysis for variances was done manually.

**Table 1:** Primer sequences for amplification

Amplicon	Forward primer	Reverse primer
Exon1 & 2	CGCTCTCCCTCCATGTTAGC	TTCCCCCTACCCCTTTGTGG
Exon3	GAAATCATTGGCACTGCCTG	AGCTGTTTAAGACCATGAGCC
Exon4	AGCTTTGAGAGGCTGTGGTC	TCTCTGGTGGTGGAGAGTCC
Exon5	ATCTTATGAGCTTGGCAGGG	GAAAAGGTTACAAACATTCCCC
Exon6	GTCTGGGAAGGTGTGATCGG	TGCCAGATATCACTGGGGTC
Exon7	GGGAAAGGGTTTAAGTCCCC	TGGAGTAATGATGGAACAGGG
Exon8	CCACCAATCCTCTCTATCTTGG	GCTTACTTCCTTTCTTCTGAGCG
Exon9	CACCCCAACTTCTCTCATGC	TAGCAAAGATGCCCTGTTCC
Exon10	CACTGGGCAGTGTTTGAATG	AGGTAGATCTTGGCGGATTC
Exon11 & *MIR32–5pa* bs	TTCTCTCTGGCATCGTGTGC	ACGCCTATTTTCTCTTGCATCT
*MIR198* & *MIR27a* bs	GAAAGACACAGGACTAACTGT	CCACTATCTGCCAGAGGAGCA
*MIR206* bs	CACTGGATCTTAACAGATGC	CTTCAAACTCCACTTGTTCAGT

Note

Sequence of the forward and reverse primers used to analyze the coding region of *FSTL1* locus (NM_007085.5); bs: binding site.

### 2.4 Copy number variation analysis

**Figure 1:**
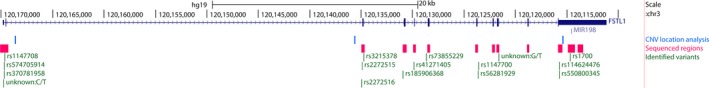
Representation of Fstl1 gene. The Fstl1 locus according to human genome GRCh37/hg19. In graph is in order indicated: scale and the genome location in chr3 (black), the FSTL1 gene (dark blue), the miRNA198 (red), the 3 regions used for copy number variation qPCR (light blue), the regions sequenced for the Sanger analysis (pink) and the identified SNP (green)

Using the primer sequences listed in Table 2, copy number variation (CNV) analysis was performed. Three regions of the *FSTL1* locus were amplified with triplicate measurements (Figure 1). As a reference, a genomic DNA region (Chr3q26.2) in which no known copy number variations occur in healthy controls was amplified (http://exac.broadinstitute.org/gene/ENSG00000206120). Quantitative PCR (qPCR) was performed using the LightCycler 480 (Roche) and the LightCycler 480 SYBR Green I Master (Roche). The qPCR data were analyzed using the LinRegPCR program (Ruijter et al., 2013).

**Table 2:** Primer sequences for copy number variation (CNV)

*FSTL1* CNV	Forward primer	Reverse primer
Start of Intron 2	GCGATGCTAAGGGTGTGGTT	GGGAGAAGAAATGGAATGCACAT
End of Intron 2	CCCATGTCTGCCACTGTCTT	CACACGTCTGAACAGGGGAA
3´‐UTR	GCGTGGATGCTGGAGGTCTG	AACTTAGCAGGGTGTCCCCGGAG
Chr3q26.2	AAGACCTCTTTCTACCACATGGT	AAATCTGAAGAAGCCACGCTT

Note

Sequences of the forward and reverse primers used to determine the CNV in the reference region and in three different regions of *FSTL1*.

### 2.5 Statistical analysis

To compare minor allele frequency between the control population and the selected patient groups, a z‐test analysis was performed. Within patients with cardiac defects, association of a specific genotype with a particular diagnosis was performed using Fisher´s exact test. Differences with p < 0.05 were considered statistically significant.

## 3 RESULTS

### 3.1 Clinical data

Based on the phenotype of the *Fstl1* KO mice, we selected 15 patients with unexplained skeletal dysplasia, of which 12 have campomelic dysplasia, 2 have small patella syndrome, and 1 with BILU. Moreover, when analyzing conditional KO mice, cardiac abnormalities have become apparent due to deletion of *Fstl1* from the endocardial/endothelial lineage. Based on this observation, we selected an additional group of 54 patients from the CONCOR biobank having abnormalities similar to the phenotype of *Fstl1* KO and *Fstl1* endoKO mice, but with an unknown genetic cause. Of these patients, 39 showed congenital abnormalities related to either the mitral or the tricuspid valve and 16 showed, besides congenital cardiac defects also kyphoscoliosis, skeletal and lung defects. A summary of the mouse phenotypes, congenital malformations, and the number of patients in each category are shown in Table 3.

**Table 3:** Summary of clinical and phenotypical data

Malformation	*Fstl1* KO	*Fstl1* endoKO	# Skeletal Dysplasia patients (*n* = 15)	# Congenital heart defect patients (*n* = 54)
Skeletal defects				
Kyphoscoliosis	+	‐	12	16
Bending of long bones	+	‐	12	12
Digit malformation	+	‐	13	‐
Small/Absent patella	+	‐	2	‐
Valves defects				
Mitral valve	‐	+	‐	39
Tricuspid valve	‐	+	‐	19
Aortic valve	‐	‐	‐	14
Pulmonary valve	‐	‐	‐	12
Other cardiac defects				
Enlarged heart	+	+	‐	3
Atrioventricular septum	‐	‐	‐	1
Atrial septum	‐	‐	‐	7
Ventricular septum	‐	‐	‐	8
Outflow tract	‐	‐	‐	15
Conduction anomalies	ns	+	‐	23
Other defects				
Lung	+	+	12	12
Renal	+	ns	13	2

Note

ns, not studied. Summary of the congenital anomalies observed in *Fst1* KO mice, *Fstl1* endoKO mice and the 69 selected patients.

### 3.2 *FSTL1* copy number variation

To investigate whether copy number variations, either duplication or deletions, were present in the *FSTL1* locus in our patient groups, we used qPCR with three different primer sets for three loci, effectively covering the entire genomic locus of *FSTL1* in combination with the reference region Chr3q26.2 (Table 2 and Figure 1)*. *Control DNA of 6 randomly selected unaffected healthy individuals was taken as reference. One out of the 15 unexplained skeletal dysplasia patients displayed an abnormal copy number variation. To follow‐up and confirm these findings, we attempted to perform an array CGH. However, an initial quality check showed that the DNA was too degraded to undergo array CGH, likely explaining the spurious qPCR findings in *FSTL1*. In the 54 patients with congenital heart disease, we did not find any CNV in *FSTL1*.

### 3.3 Identification of two novel intronic variants

*FSTL1* encodes a 308‐amino acid protein with an *N*‐terminal signal peptide of 20 amino acids. *FSTL1* is composed of 11 exons, the first exon encodes the noncoding 5’UTR, and exon 11 encodes a small part of coding sequence and a long 3’UTR. In this 3’UTR, *MIR198* is encoded and 3 functionally validated binding sites for miRNAs are present (Figure 1). Using PCR, all the *FSTL1* coding exons and their immediately flanking regions, the *MIR198* and the miR‐binding sites, were amplified and sequenced. We found 16 nucleotide variations in our patients compared to the human reference genome (Hg19). We did not identify any protein‐altering variants in any of our patient groups. Only variations in the noncoding intronic regions of *FSTL1* were found (Table 4 and shown in Figure 1). Out of the 16 identified variations, 14 are known polymorphisms and 2 are novel variants not present in over 120 k exomes (http://gnomad.broadinstitute.org/). The first novel variant is located in intron 1 (chr3:120,169,715); it is a C > T nucleotide change in a patient with atrioventricular and outflow tract valves defects. This portion of the *FSTL1* gene is an epigenetic dense region and likely to be a part of the promoter of Fstl1, based on the integration of multiple epigenetic marks (van Duijvenboden, Boer, Capon, Ruijter, & Christoffels, 2016). The second novel variant is positioned at chr3:120,121,556 and is a G > T nucleotide change located in intron 9, in a patient with campomelic dysplasia. In this region, there is no evidence of evolutionary conservation or regulatory regions. Both SNPs are outside the splicing donor/acceptor sites.

**Table 4:** Identified variants

RS number	Nucleotide change	Position Hg19	Location
Unknown	C/T	chr3:120169714	Intronic (30 bp downstream exon 1)
rs370781958	C/A	chr3:120169697	Intronic (47 bp downstream exon 1)
rs574705914	C/T	chr3:120169668	Intronic (76 bp downstream exon 1)
rs1147708	C/T	chr3:120169641	5’UTR
rs2272516	C/A	chr3:120134901	Intronic (27 bp upstream exon 3)
rs2272515	T/C	chr3:120134883	Intronic (9 bp upstream exon 3)
rs3215378	‐/G	chr3:120134720	Intronic (49 bp downstream exon 3)
rs185906368	C/T	chr3:120130874	Intronic (44 bp upstream exon 4)
rs41271405	T/C	chr3:120129860	Intronic (29 bp upstream exon 5)
rs73855229	C/T	chr3:120128618	Intronic (109 bp upstream exon 6)
rs56281929	T/G	chr3:120123572	Intronic (128 bp downstream exon 7)
rs1147700	A/C	chr3:120123556	Intronic (140 bp downstream exon 7)
Unknown	G/T	chr3:120121556	Intronic (99 bp downstream exon 9)
rs550800345	C/T	chr3:120115713	3’UTR
rs114624476	T/C	chr3:120115683	3’UTR
rs1700	C/T	chr3:120114637	3’UTR

Note

Summary of the different nucleotide variations found in 69 selected patients.

### 3.4 Differences in frequency of intronic variants

Comparison of the minor allele frequency (MAF) of the intronic variants identified in our patients with those of 1,000 genomes project (www.ensembl.org) revealed that 3/11 variants identified in the skeletal dysplasia group, and 5/10 in the congenital heart defect patients, differed significantly (Table 5). However, none of these variants have been associated with a pathological function. Due to the variability in the phenotype observed in these patients, we tried to correlate the clinical defects reported in our patients, with the identified variants. We observed an association between variation rs2272515 and kyphoscoliosis (*p* = 0.0487), and a trend (*p* = 0.0508) with pulmonary valve defects.

**Table 5:** Variant frequency

RS number	MAF control 1000G (2504)	MAF skeletal dysplasia (15)	p value	MAF congenital heart defect (54)	p value
Unknown	‐	‐	‐	0.01 (1)	2.7E−05*
rs370781958	0.01 (43)	‐	‐	0.01 (1)	0.94
rs574705914	<0.01 (17)	‐	‐	0.01 (1)	0.31
rs1147708	0.16 (532/87)	0.07 (1)	0.09	0.06 (6)	0.01*
rs2272516	0.09 (142/19)	0.27 (4)	4.5E−3*	0.01 (1)	0.14
rs2272515	0.40 (917/251)	0.40 (6)	0.31	0.61 (21/10)	3.1E−07*
rs3215378	0.28 (914/251)	0.40 (6)	0.32	‐	‐
rs185906368	0.05 (209/17)	0.07 (1)	0.70	0.07 (7)	0.44
rs41271405	0.04 (193/6)	0.07 (1)	0.83	‐	‐
rs73855229	0.02 (88/3)	0.07 (1)	0.56	‐	‐
rs56281929	0.04 (197/6)	0.07 (1)	0.82	‐	‐
rs1147700	0.3 (931/323)	0.27 (4)	0.03*	‐	‐
Unknown	‐	0.07 (1)	1.2E−4*	‐	‐
rs550800345	<0.01 (1)	‐	‐	0.01 (1)	2.4E−06*
rs114624476	<0.01 (15)	‐	‐	0.02 (2)	5.5E−3*
rs1700	0.08 (381/22)	0.13 (2)	0.89	0.08 (9)	0.95

Note

Data on the frequency and statistics of the identified nucleotide variations. If available, the reference of the single nucleotide polymorphism identification number (RS) is given in the first column. The minor allele frequency (MAF) of controls as identified in the 1000 genome project, as well as in the two patient groups, is shown. In brackets, the number of heterozygous and, when applicable, homozygous individual is indicated.

* *p*‐values less than 0.05 were considered significant.

## 4 DISCUSSION

Deletion of *Fstl1* in mice results in skeletal and respiratory abnormalities (Sylva et al., 2011) and conditional deletion from endocardial/endothelial lineage shows profound AV valve defects (Prakash et al., 2017). To the best of our knowledge, homozygous loss‐of‐function pathogenic variants in *FSTL1* have never been reported, and heterozygous ones have only been described in 35 individuals. In line with these findings, the estimated probability of loss‐of‐function intolerance of *FSTL1* is 0.96 (http://exac.broadinstitute.org/gene/ENSG00000163430), comparing the exomes of over 120,000 humans. This suggests that pathogenic variations are not tolerated in the coding region of *FSTL1* gene. This finding is not surprising considering that complete disruption of *Fstl1* (*Fstl1* KO) in mice results in death of the newborns due to their inability to breathe. Furthermore, these *Fstl1* KO mice display major skeletal defects, including bending of the spine and long bones, and an enlarged heart (Sylva et al., 2011). Conditional removal of *Fstl1* from the endocardial/endothelial cell lineage (*Fstl1* endoKO) results in mice that die between 2 and 4 weeks after birth with heart failure with preserved ejection fraction and dysfunctional valves (Prakash et al., 2017). The mitral valve displays a myxomatous phenotype with mitral valve prolapse and regurgitation. It should be noted, that in different genomewide association studies (GWAS), *FSTL1* has never been associated with mitral valve prolapse (Dina et al., 2015; Freed et al., 2003; Nesta et al., 2005). Conditional removal of *Fstl1* from the cardiomyocytes (Shimano et al., 2011) or from the cardiac fibroblasts (S100A or Col1a1) (Maruyama et al., 2016) did not result in any gross abnormalities in mice.

To identify potential carriers of variations in the *FSTL1* gene, we compared the phenotypes of the *Fstl1* KO and endoKO mice to rare congenital malformations reported in humans. We identified a total of 69 patients comprising the following groups: (a) skeletal defects, such as campomelic dysplasia, small patella syndrome and BILU syndrome, and (b) patients with cardiac anomalies, valve defects in the majority, in combination with or without kyphoscoliosis. Importantly, in none of these selected patients, a known genetic defect was reported. Analysis of the coding sequence with respect to the copy number of the *FSTL1* gene revealed no genetic abnormalities in either of these 69 patients. In 52 patients, a total of 16 variations were found. These variations were only found in the noncoding region of the *FSTL1* gene. Eleven were found in patients with skeletal dysplasia, and three of these SNP showed significant differences in minor allele frequency (MAF) compared to control populations. Ten of these SNPs were found in patients from the cardiac anomalies group, and five of these SNP showed significant differences in MAF compared to control populations. The intronic variant rs2272515 significantly correlates with kyphoscoliosis in our population. This variant is located 9 bp upstream exon 3 in the splice acceptor sequence and could, provided it is biologically functional, affect mRNA maturation causing a truncated form of the protein. In the congenital heart disease patients, 3 SNPs are located in the 3’UTR of *FSTL1*. The location of these SNPs was not in the encoded miRNA or in either of the validated miRNA‐binding site. To evaluate whether these SNPs might affect an unknown microRNA‐binding site, www.microRNA.org and www.targetscan.org were used to predict miRNA‐binding sites in the region of the identified SNP. This analysis revealed that SNP rs1700 overlapped with the predicted binding site for *MIR154*, which is known to promote myocardial fibrosis (Dong, Liu, & Wang, 2018) and regulating the metastatic behavior of nonsmall lung cancer cells (Liu et al., 2018). As both biological effects of *MIR154* are among the biological functions of *FSTL1* (for review see (Mattiotti et al., 2018)), this might be an inroad for further analysis. However, due to the relative small patient group, the frequency of this variant is not significantly different between the patients and control groups.

In conclusion, we did not find any pathogenic variations in the coding region of the *FSTL1* gene in 69 patients suffering from rare, and genetically unresolved, skeletal or cardiac disorders, that resembled the phenotypes observed in complete or conditional *Fstl1* KO mice. However, we identified 8 intronic *FSTL1* variants that significantly differed from control populations in minor allele frequency, and found two novel/unique intronic variants. Taken together, we conclude that pathogenic variations in *FSTL1* are likely not responsible for skeletal or atrioventricular valve anomalies in humans.

## CONFLICT OF INTEREST

The authors state that there are no conflicts of interest to disclose.
